# The room temperature crystal structure of a bacterial phytochrome determined by serial femtosecond crystallography

**DOI:** 10.1038/srep35279

**Published:** 2016-10-19

**Authors:** Petra Edlund, Heikki Takala, Elin Claesson, Léocadie Henry, Robert Dods, Heli Lehtivuori, Matthijs Panman, Kanupriya Pande, Thomas White, Takanori Nakane, Oskar Berntsson, Emil Gustavsson, Petra Båth, Vaibhav Modi, Shatabdi Roy-Chowdhury, James Zook, Peter Berntsen, Suraj Pandey, Ishwor Poudyal, Jason Tenboer, Christopher Kupitz, Anton Barty, Petra Fromme, Jake D. Koralek, Tomoyuki Tanaka, John Spence, Mengning Liang, Mark S. Hunter, Sebastien Boutet, Eriko Nango, Keith Moffat, Gerrit Groenhof, Janne Ihalainen, Emina A. Stojković, Marius Schmidt, Sebastian Westenhoff

**Affiliations:** 1 Department of Chemistry and Molecular Biology, University of Gothenburg, 40530 Gothenburg, Sweden; 2 Department of Biological and Environmental Sciences, University of Jyvaskyla, 40014 Jyvaskyla, Finland; 3 Faculty of Medicine, Anatomy, University of Helsinki, 00014 Helsinki, Finland; 4 Nanoscience Center, Department of Physics, University of Jyvaskyla, 40014 Jyvaskyla, Finland; 5 Center for Free-Electron Laser Science, Deutsches Elektronen-Synchrotron DESY, D-22603 Hamburg, Germany; 6 Department of Biological Sciences, Graduate School of Science, The University of Tokyo, 7-3-1 Hongo, Bunkyo-ku, 113-0033, Tokyo, Japan; 7 Nanoscience Center, Department of Chemistry, University of Jyvaskyla, 40014 Jyvaskyla, Finland; 8 School of Molecular Sciences, Arizona State University, 85287 Tempe, Arizona, USA; 9 Australian Research Council Centre of Excellence for Advanced Molecular Imaging, La Trobe Institute for Molecular Science, La Trobe University, 3086 Melbourne, Victoria, Australia; 10 Physics Department, University of Wisconsin, WI 53211 Milwaukee, USA; 11 Linac Coherent Light Source, SLAC National Accelerator Laboratory, 2575 Sand Hill Road, Menlo Park, CA 94025, USA; 12 RIKEN SPring-8 Center, 1-1-1 Kouto, Sayo-cho, Sayo-gun, 679-5148 Hyogo, Japan; 13 Biodesign Institute, Arizona State University, 85287 Tempe, Arizona, USA; 14 Center for Applied Structural Discovery, Arizona State University, 85287 Tempe, Arizona, USA; 15 Department of Physics, Arizona State University, 85287 Tempe, Arizona, USA; 16 Department of Biochemistry and Molecular Biology, University of Chicago, 60637 Chicago, Illinois, USA; 17 BioCARS, Argonne National Laboratory, 60439 Argonne, Illinois USA; 18 Nanoscience Center, Department of Biological and Environmental Sciences, University of Jyvaskyla, 40014 Jyvaskyla, Finland; 19 Department of Biology, Northeastern Illinois University, 5500 North St. Louis Avenue, 60625 Chicago, Illinois, United States

## Abstract

Phytochromes are a family of photoreceptors that control light responses of plants, fungi and bacteria. A sequence of structural changes, which is not yet fully understood, leads to activation of an output domain. Time-resolved serial femtosecond crystallography (SFX) can potentially shine light on these conformational changes. Here we report the room temperature crystal structure of the chromophore-binding domains of the *Deinococcus radiodurans* phytochrome at 2.1 Å resolution. The structure was obtained by serial femtosecond X-ray crystallography from microcrystals at an X-ray free electron laser. We find overall good agreement compared to a crystal structure at 1.35 Å resolution derived from conventional crystallography at cryogenic temperatures, which we also report here. The thioether linkage between chromophore and protein is subject to positional ambiguity at the synchrotron, but is fully resolved with SFX. The study paves the way for time-resolved structural investigations of the phytochrome photocycle with time-resolved SFX.

Phytochromes sense ambient light levels in plants, fungi, and bacteria. The proteins detect light and trigger intracellular signalling cascades, which regulate many light-dependent phenotypes. Examples include shade avoidance and seed germination in plants^1^,^2^, the control of the abundance of photosynthetic proteins in cyanobacteria^3^, and the control of carotenoid expression in bacteria^4^. In bacteria, the proteins often function as histidine kinases in a two-component signalling system^5^.

Phytochromes share a modular domain architecture. The widely conserved photosensory core module usually consists of PAS (PER, ARNT, SIM), GAF (cGMP phosphodiesterase, adenylate cyclase, FhlA), and PHY (Phytochrome-specific GAF related) domains^5^. The PAS and GAF domains hold a tetrapyrrole bilin chromophore, represented by phytochromobilin, phycocyanobilin, or biliverdin in plants, cyanobacteria, or bacteria, respectively. The chromophore is covalently attached via a thioether linkage to a conserved cysteine in either the PAS domain (bacteria) or GAF domain (cyanobacteria and plants) (Fig. 1).

Upon light absorption by the chromophore, a sequence of structural changes is initiated, which shuttles the protein from a red light-absorbing state (Pr) to a far-red light-absorbing state (Pfr) or vice versa. These structural changes involve at least a *Z*-to-*E* isomerization of the C15 = C16 double bond of the chromophore with associated changes of the chromophore-binding pocket^6^,^7^,^8^,^9^ and refolding of the so-called PHY-arm^6^,^7^,^10^,^11^. It is unclear how these changes extend from the photosensory core into the output domains. Furthermore the details of the isomerization reaction and the sequence of how the conformational changes arise remain poorly understood.

Conformational heterogeneity of the chromophore has been suggested to play an important role in the photocycle. Two Pr-state conformations have been detected for a cyanobacterial phytochrome in solution with NMR spectroscopy^12^. This finding is contrasted by the crystal structures of phytochromes, which only show one chromophore conformation^6^,^7^,^13^,^14^, at resolutions approaching 1 Å (this work and ref. 9). It is not clear whether the crystal packing, or cryogenic temperature at which crystallographic data are recorded, favours only one conformation, or whether the crystal structures truly reflect the solution structures.

SFX is an attractive method for resolving the structural changes in light-activated proteins^15^. In SFX, diffraction data are collected from a stream of microcrystals in a liquid jet flowing across the X-ray focus. Data from thousands of individual microcrystals, each exposed only to a single femtosecond X-ray pulse, are sorted, indexed, and merged into one complete set of reflection intensities^16^,^17^. Thus it becomes possible to expose the crystals to an optical laser pulse prior to data collection. Structural snapshots of the protein at defined stages of the photoreaction can be recorded^15^,^18^. In this paper we report the crystal structure for the chromophore-binding domains (PAS-GAF) of *Deinococcus radiodurans* recorded by SFX. Crystallographic data are collected at room temperature and the crystals are not subject to significant radiation damage at our resolution^19^,^20^.

## Results

### Crystallisation and data collection

Modified crystallisation conditions were developed to yield good quality macrocrystals. The PAS-GAF fragment of the *D. radiodurans* phytochrome was crystallised in essentially the same conditions as reported elsewhere^13^, but the cryoprotectant 2-methyl-2, 4-pentanediol was added directly to the crystallisation buffer (see methods). Those changes resulted in the protein packing as monomers instead of dimers in contrast to previously published conditions^13^. The space group C121 and cell dimensions were highly similar to the crystals from monomeric PAS-GAF^21^. We recorded diffraction data at cryogenic conditions at the European Synchrotron Radiation Facility (ESRF) and solved the structure to 1.35 Å resolution by molecular replacement with 4Q0H^9^ as a search model. We refer to this structure as “LowT”.

A protocol for the preparation of microcrystals suitable for SFX was developed based on the conditions described above. The microcrystals were grown in batches of 0.5 mL in a microcentrifuge tube at 10 degrees Celsius (see methods). The typical crystal size was 5–10 μm with a narrow size distribution as judged by visual inspection with a microscope (see Fig. 2a). Figure 2c compares the absorption spectra of the protein in solution and in microcrystal suspensions. The microcrystal spectrum is highly similar to soluble one, except for a 5 nm red shift and a small shoulder at the far-red part of the 700 nm peak (Q band).

To record SFX data from these crystals, the suspensions were injected into a vacuum chamber using a gas dynamic virtual nozzle^22^ at the CXI instrument of the Linac Coherent Light Source (LCLS)^23^. The stream of crystals was exposed to femtosecond X-ray pulses of approximately 1.8 mJ/pulse at 9.5 keV. 114409 hits were identified using Cheetah^24^ and 90428 frames were indexed and merged using CrystFEL version 0.6.1 + c9c7^25^. The structural model was solved using Phaser with 4Q0H^9^ as a search model. We refer to this structure as “SFX”.

### Comparison of room temperature and cryogenic structure

**Figure 1:**
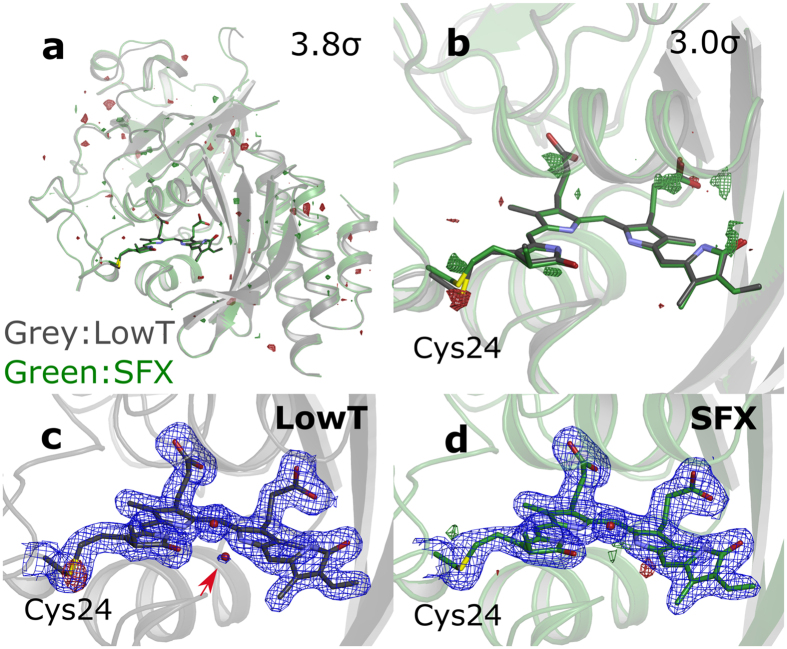
Comparison of the crystal structures for the PAS-GAF fragment from bacteriophytochrome from D. radiodurans at cryogenic (LowT) and room (SFX) temperatures. The datasets are truncated at 2.1 Å resolution in all panels. (**a**) Superimposed SFX (green) and LowT (grey) structures plotted together with the Fo(SFX)-Fo(LowT) difference map (red: negative, green: positive) at 3.8 σ. The overall R-factor for difference map is 24.5%. (**b**) The same representation zoomed into the chromophore region. The Fo(SFX)-Fo(LowT) difference map is contoured at 3.0 σ. (**c**) The LowT structure and the corresponding maps (2Fo-Fc, blue at 1.3 σ and Fo-Fc difference map at 3.3 σ). The extra water only present in the LowT structure is indicated by a red arrow. (**d**) The SFX structure and the corresponding maps (2Fo-Fc and Fo-Fc maps, contour levels as in c).

Figure 1 compares the LowT (PDB code 5K5B) and SFX (PDB code 5L8M) structures. For the SFX structure, X-ray data were recorded at room temperature and appear essentially free of radiation damage at our resolution. This is a consequence of the “diffraction before destruction” principle, where each crystal is exposed only once to a single X-ray pulse with femtosecond duration^19^. Conventional crystallography is typically performed at cryogenic temperatures and changes may occur upon cooling^26^. Also, radiation damage typically occurs, depending on the X-ray dose. Overall, we find excellent agreement between our structures at cryogenic and ambient temperatures. Figure 1a shows the difference electron density (Fo(SFX) - Fo(LowT)) at 3.8 σ contour level for the whole protein. The positive and negative peaks are mostly localized to the outer parts of the globular domains. This is expected, since thermal motions are likely to be more significant at these positions. However, very few difference density peaks are observed on the backbone atoms and on the chromophore, indicating close agreement between the two structures.

Figure 1b demonstrates the Fo(SFX) - Fo(LowT) map of the biliverdin chromophore and its close surroundings. At 3σ the only associated positive and negative electron densities are observed close to the thioether bond of the chromophore. This indicates a different position of the thioether linkage in the LowT compared to the SFX structure. It has been shown that flash-cooling can introduce packing and local structural perturbations and it is likely that the differences in the Fo maps reflect such effects^26^. Moreover, this part of the structure is not uniquely resolved in the LowT data, and the associated negative peak are observed in the Fo-Fc maps (Fig. 1c). However, the Fo-Fc map of the SFX structure (Fig. 1d) does not indicate any discrepancy between data and model and a unique covalent bonding geometry was refined. The diffuse electron density in the LowT structure (Fig. 1c) could be due to radiation damage or to alternate conformations of the chromophore-protein bond. In any case, our SFX structure does not show this ambiguity. The SFX structure confirms, to a resolution of 2.1Å, that a single chromophore geometry is present in the crystals.

We also note that the LowT data support a water molecule with partial occupancy close to Tyr263 (arrow in Figs 1c and 3). The water is hydrogen-bonded to the A-ring carbonyl, the pyrrole water and the functionally important Asp207. The electron density from SFX data did not support the modelling of the water molecule. We postulate that this water either exchanges rapidly with the solvent surroundings or that it is expelled by higher overall thermal motion of the surrounding atoms at room temperature.

### Structural differences and chromophore structural properties

In Fig. 3 our structures are compared to other PAS-GAF structures. On the one hand, our structures agree well with structures of a monomeric variant (4IJG) of PAS-GAF, which has three mutations to block dimerization (Fig. 3a)^21^. On the other hand, the positions of the backbone atoms, the chromophore and its binding pocket differ from a certain group of structures. These structures originate from crystals formed of proteins with a mutation (Y307S) to aid crystallisation which, by altering the crystal contacts, results in a different crystal packing (see Fig. 3a,b)^8^,^9^.

**Figure 2:**
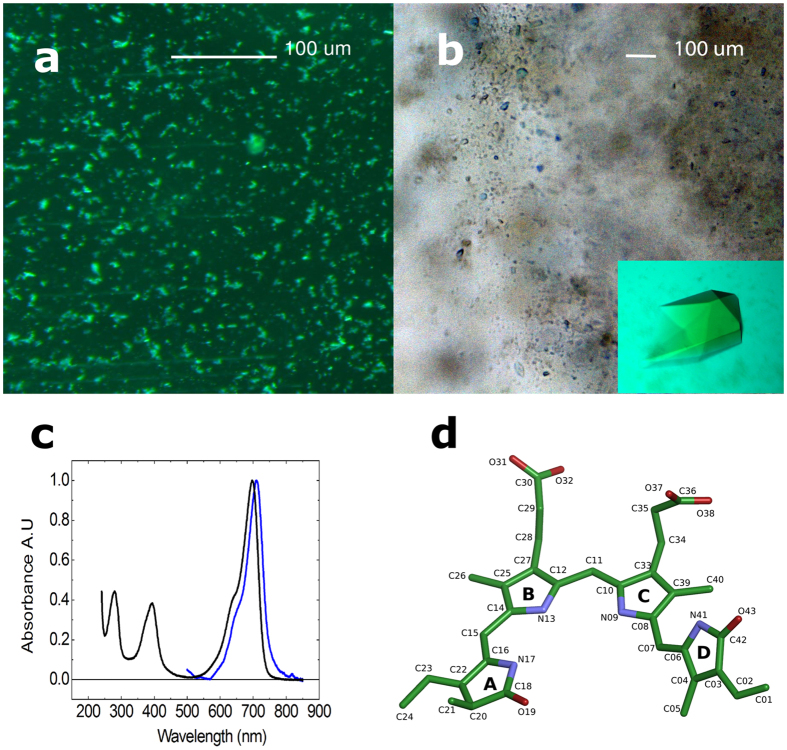
Microcrystallisation of PAS-GAF. (**a**) Micrograph of microcrystals produced by direct crystallisation in microcentrifuge tubes, and (**b**) the microcrystals produced by crushing macrocrystals (inset) with seed beads. (**c**) Absorption spectra of PAS-GAF in solution (black) and microcrystals (blue). (**d**) Structure and atom names of the bilverdin cofactor.

**Figure 3:**
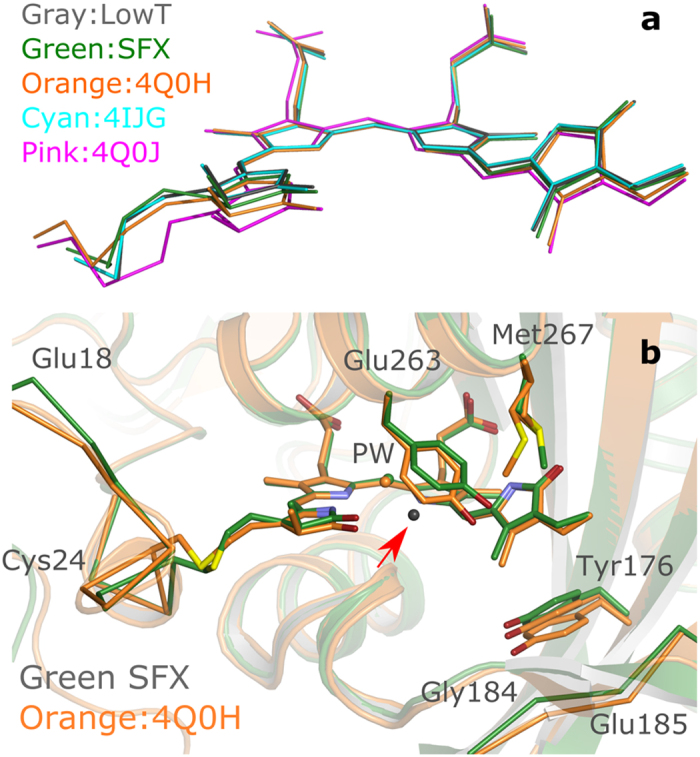
Comparison of LowT and SFX structures to earlier published PAS-GAF structures. (**a**) Comparison of the biliverdin conformation and cystein24 thioether linkage of LowT and SFX structures with previously reported PAS-GAF structures 4IJG^21^, 4Q0H^9^, and the PAS-GAF-PHY structure 4Q0J^9^. 4Q0H has very similar conformation around the thioether bond compared to 2O9C and 2O9B^8^, which are not displayed for clarity. (**b**) Comparison of the SFX structure and 4Q0H^9^ PAS-GAF-structures reveals differences in the D- and A-ring orientation, the thioether linkage and the positions of Tyr263, Met267, Tyr173 and the residues 17–25 and 184–186. The grey water (marked by red arrow) is only observable in the LowT structure (see text for details).

In particular, we found that the D-ring of the biliverdin chromophore is more planar in our structures (dihedral_N09,C08,C06,C04_ = 64.8 degrees compared to 73.4 degrees in 4Q0H, see Fig. 2d for biliverdin nomenclature) and that the Tyr263 and Tyr176, which sandwich the D-ring, adjust accordingly (Fig. 3b). From Tyr176 these structural differences translate to a slight upward shift of the backbone of residues 184–186. Tyr263 shifts away from the pyrrole water, which also makes room for the extra water between the pyrrole water and the phenol ring, which is observed by electron density in the LowT structure (Fig. 1c). Furthermore, Met267, which is a direct neighbour of Tyr263 appears in a different position. The density for this residue appears diffuse in our LowT structure (data not shown) and the same residue has been modelled by others with multiple conformations^9^. In the new SFX structure, the electron density for this residue does not support multiple conformations; hence the diffuse electron densities in the LowT structures may arise from radiation damage. Structural differences between the new structures and 4Q0H are also seen in the residues flanking Cys24 and the thioether linkage (Fig. 3b). In fact, many published wild-type PAS-GAF structures show different conformations of the Cys24 and the A-ring of the chromophore, indicating enhanced conformational flexibility in this region (Fig. 3a).

### Method and structural results are enforced by additional experiment

**Figure 4:**
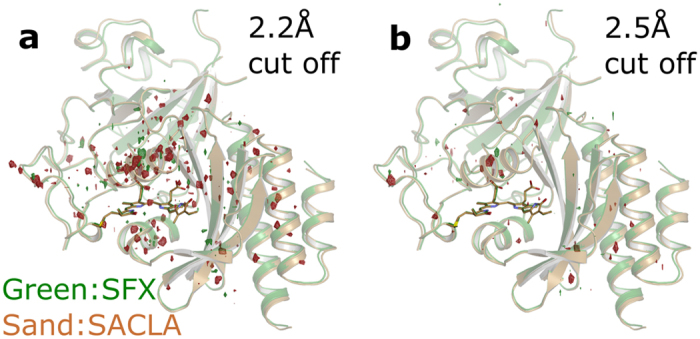
Structural overlay of the SFX (green) and SACLA (sand) structures plotted together with the Fo(SFX)-Fo(LowT) difference map (red: negative, green: positive) at 3.8 σ. (**a**) data truncated at 2.2 Å (overall R-factor 14.6%) and (**b**) data truncated at 2.5 Å (overall R-factor 11.6%).

Finally, we report a second method to generate micron-sized phytochrome crystals for performing SFX experiments. To this end, macrocrystals were crushed with seed beads by vortexing in a microcentrifuge tube. Microscope images revealed that the crystal slurry had a broad size distribution even after filtering through a 20 μm filter and contained crystals sizes ranging from 5 μm to 30 μm (see Fig. 2b). These sets of crystals were dispersed in grease^27^ and exposed to short pulses of X-rays at 7 keV at the SPring-8 Angstrom Compact free electron LAser (SACLA). 30146 hits were recorded in 3 hours of beamtime of which 20014 were indexed using CrystFEL^25^. The structure shows overall good agreement with the other structures reported in this paper (see Fig. 4). When comparing the difference electron density map (SFX-SACLA) at 2.5 Å resolution, only very few difference peaks are observed (Fig. 4b). At higher resolution the data recorded at SACLA starts to diverge from the SFX data set (Fig. 4a). This is likely due to the fact that less frames with high resolution data were recorded at SACLA, which can be traced back to the broad size distribution of the crystal preparation.

## Discussion

Here we report the crystal structure in a new crystal form of the phytochrome PAS-GAF domains from *D. radiodurans.* Compared to other structures of the same protein fragment, these crystal conditions did not require monomerizing mutations^28^ or mutations to stabilise the crystal packing^8^,^9^. The 1.35 Å resolution to which the LowT structure has been determined is the highest reported so far for a wild-type PAS-GAF fragment. Importantly, the LowT structure corroborates the SFX structures. The comparison is very direct, because the crystallisation conditions for the SFX and conventional crystallography were identical (SACLA) or highly similar (LCLS). More generally, this implies that a large body of structural conclusions, which were drawn from crystal structures obtained at cryogenic temperatures, are also valid at ambient conditions^6^,^7^,^9^,^10^,^14^,^29^.

**Figure 5:**
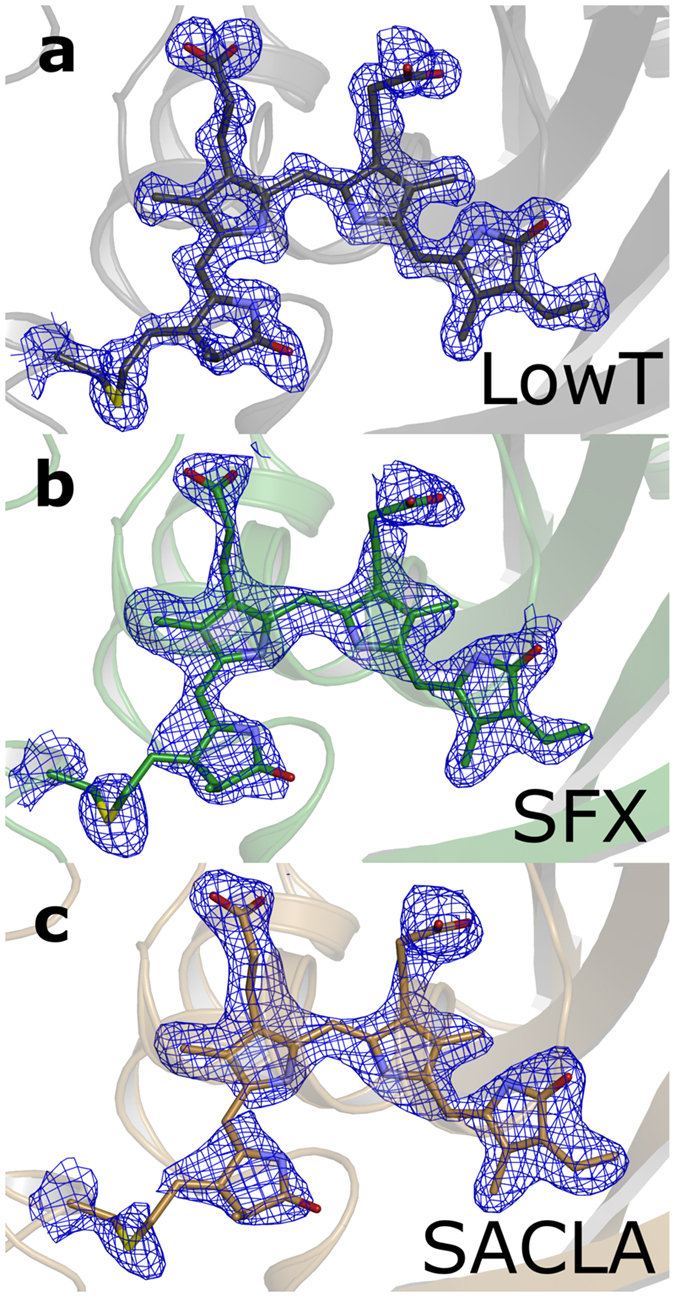
The omit maps of the chromophore (blue) contoured at a 1.5 σ. (**a**) The LowT structure in grey (**b**). The SFX structure in green. (**c**) SACLA in sand.

Except for the ambiguity in the thioether bond in the LowT data, the SFX and LowT data support only one chromophore conformation (Fig. 3a). This is further confirmed by composite omit maps (Fig. 5). This finding is in agreement with other reported structures, which all found only one conformation^6^,^7^,^8^,^9^,^13^,^30^. However, the conformation of the chromophore and its thioether linkage reported here differs from the previously solved structures of the same phytochrome fragment (Fig. 3)^8^,^9^,^13^. These crystals had a different packing and thus the differences in conformation are most likely introduced by packing effects. The structures presented in this paper confirm that the chromophore and protein can, in principle, adopt an alternative conformation. It is therefore likely that the protein in solution adopts, at least, both conformations. These findings are in qualitative agreement with the previous reported results that a cyanobacterial phytochrome in Pr exists in at least two conformations^12^ and that the spectroscopic evolution supports heterogeneous conformations^31^.

In order to record SFX data, the microcrystals have to be delivered to the beam. There are currently two main approaches to achieve this. In one method, a buffer solution is used as the carrier medium to generate a thin jet that propagates at high flow rates^22^. In the second approach, viscous media such as grease or lipid/water mixtures, are used, which facilitates significantly slower flow rates^27^,^32^. The slow flow conserves protein. Here we demonstrate how phytochrome microcrystals can be used with both methods to obtain SFX crystal structures. We have developed two ways of producing phytochrome microcrystals, by crushing macrocrystals and by directly growing microcrystals in batch mode. This versatility allows upcoming SFX experiments on phytochromes to be optimised for hit rate, crystal quality and ease of crystal production. Although we have not verified the integrity of the crystals in grease spectroscopically, the SFX structures presented here confirm the structural integrity of the protein in both delivery methods.

It is currently unclear which conformational changes in the protein and the chromophore govern photo activation. To investigate this further, the microcrystals could be subjected to femtosecond time-resolved SFX. In the experiment, photo activated states are prepared by femtosecond optical laser pulses, which arrive at a defined delay time prior to the X-ray exposure. Time-resolved spectroscopy experiments of phytochromes^33^,^34^ indicate that high concentrations of the excited state can be prepared, but that the quantum yield of entering the photocycle upon return to the ground state is only on the order of 10–15%. Thus, it may be easiest to detect structural changes in the excited state, which lasts for a few hundreds of picoseconds. Observing conformational changes by crystallography also requires sufficient resolution. The resolution of 2.1 Å, which we demonstrate here, should be fine enough to detect the isomerisation reaction in the chromophore, changes in water positions, and movement of amino acids side chains. The homogeneity of the crystal size and shape will be crucial since it affects laser penetration and excitation efficiency. Therefore the development of batch crystallisation for highly homogeneous microcrystals is important. The study presented here shows that the excellent diffraction data of the resting states of phytochromes can be recorded by SFX and encourages time-resolved SFX experiments.

## Methods

### Protein purification

Wild-type chromophore-binding domain (PAS-GAF) from *Deinococcus radiodurans* was expressed and purified following methods already described elsewhere^10^,^35^. Briefly, (His)6-tagged PAS-GAF was produced in E.coli strain BL21 DE3. The protein production was induced with IPTG, and biliverdin was added to the lysed cells to be incorporated overnight. The holoprotein was then purified with Ni-NTA affinity chromatography (HisTrap, GE healthcare) and size-exclusion chromatography (HiLoad 16/600 Superdex GE healthcare). The purified protein was concentrated to 25–30 mg/ml in buffer (30 mM Tris, pH 8.0) and was flash-frozen in liquid nitrogen.

### Crystallisation

All crystals were set up and grown in complete darkness or under green safe light. Before crystallisation, the protein sample (PAS-GAF) was thawed and filtered with 0.2 μm centrifugal filter units. Vapour diffusion crystallisation was performed, where 20 mg/ml protein in buffer (30 mM Tris, pH 8.0) was mixed in a 1:1 ratio with reservoir solution using conditions previously described (67 mM Sodium acetate pH 4.95, 3.3% PEG 400, 1 mM DTT)^13^, except for 30% 2-methyl-2, 4-pentanediol, which was included directly in the reservoir buffer. 2 μl hanging drops were equilibrated against 800 μl of reservoir solution. After 48 h incubation at room temperature, the formed crystals were flash-frozen in liquid nitrogen in cryoloops for crystallographic data collection.

For batch crystallisation of microcrystals, which was used in the LCLS experiment, 50 μl protein (25 mg/ml) were mixed with 450 μl of reservoir solution (60 mM Sodium acetate pH 4.95, 3.3% PEG 400, 1 mM DTT and 30% 2-methyl-2, 4-pentanediol) in 1.5 ml microcentrifuge tubes followed by immediate vortexing. The tubes were then constantly mixed on a rocking table at 12 °C for ~36 h, yielding homogenous mixture of microcrystals of 10–20 μm size. The crystals were concentrated by centrifugation into a crystal concentration up to 20% to increase crystal hit rate and filtered directly before injection to minimize clogging of the jet.

For preparation of microcrystals by crushing macrocrystals, which were used at SACLA, the above vapour diffusion crystallisation conditions were used, but the macrocrystals were grown in sitting drops with 40 μl total volume. The crystal-containing drops were pooled in a microcentrifuge tube and the crystals were crushed by vortexing with seed beads (Molecular dimensions) for 30 s, after which the tubes were cooled on ice. The size of the crystals was estimated under the microscope to be ~10–20 μm, and oversized crystals were excluded with a 20 μm cutoff centrifugal filter (Partec). Crystals were concentrated and mixed with a grease (Superlube, Syncho chemical corp.) prior to loading into the syringe pump injector as described elsewhere^27^.

### Data collection and processing at the ESRF

The low temperature (LowT) macrocrystal data were acquired at beamline ID23-1 of the European Synchrotron Radiation Facility (ESRF), in a 100 K cryostream and with an X-ray wavelength of 0.980 Å. Diffraction data were processed using the XDS program package version January 10, 2014^36^. The LowT crystal belong to monoclinic space group C121 with one monomer in an asymmetric unit. The diffraction data were cut at a cross-correlation (CC1/2) value of 0.76^37^, which corresponds the high resolution limit of 1.35 Å. The statistics of data collection, processing, structure determination and refinement of all data sets are summarized in Table 1.

### Data collection and processing at the LCLS

SFX data of microcrystals were collected at the coherent X-ray Imaging CXI beamline at Linac Coherent Light Source (LCLS) using 1.8 mJ/pulse at 9.5 keV X-rays with a pulse duration of ~35 fs and a repetition rate of 120 Hz^23^. After concentrating and filtering through a 20 μm cutoff filter, the crystal suspensions were injected into the X-ray beam with gas virtual dynamic nozzles ranging from 50 μm to 100 μm diameter^22^, using a pressurized reservoir mounted on an antisettling device (available at LCLS). The flow rates of the liquid gas and shielding gas were adjusted to obtain a stable jet with a flow rate averaging around 30 μl/min. The time between filtering and injection was kept minimal to avoid excessive clogging of the reservoir and tubing.

The diffraction images were recorded with Cornell-SLAC Pixel Array detectors, consisting of 64 tiles (each 194 pixels by 185 pixels) with a pixel size of 110 × 110 μm2. A crystal “hit” was defined as an image containing a minimum of 20 diffraction peaks. Crystal hits were identified using the software package Cheetah^24^. The crystal hit rate was typically 2–5% and appeared to be limited by the tendency of the crystals to aggregate, which blocked the tubing or reservoir. The collected data were processed using Cheetah^24^ and CrystFEL^25^. The indexing rate was stable at around 70–80%. The crystals belonged to C121 space group with highly similar cell parameters to LowT crystals (Table 1).

### SFX data collection and processing at SACLA

SFX diffraction data were collected at the BL3 beamline at SACLA^38^ using 7.0 keV X-rays with a pulse duration of <10 fs and a repetition rate of 30 Hz. The syringe-pump injector was installed in the diffraction chamber filled with helium^39^. Phytochrome crystals were dispersed in grease^27^ and extruded through 110 μm diameter nozzle at a flow rate of 500 nl per minute. Diffraction patterns were recorded on a multiport CCD detector with an eight sensor module^40^. The crystal hit rate varied between 5 and 15%. The raw images were filtered and converted by Cheetah pipeline adapted for SACLA^41^ and the processing was performed as described above. For further details on number of indexed patterns etc., see Table 1.

### Structure determination and refinement

The structures were solved by molecular replacement with Phaser^42^ using the high-resolution structure of *D. radiodurans* PAS-GAF fragment 4Q0H^9^ as a search model. The models were built in Coot^43^, and structural refinement was performed with REFMAC5 version 5.8.0135^44^. In all refinements, the same topology/parameter (CIF) file for the chromophore was used as in ref. 9. In the LowT structure refinement, the X-ray matrix-weighing term of 0.5 was used. The structure was refined with anisotropic temperature factors, resulting final Rwork/Rfree of 14.2/17.0%. Ramachandran statistics of the final LowT structure had 97.8% in preferred and 2.2% in allowed regions with 0% outliers. In SFX structures (LCLS and SACLA) refinement, the weighing term of 0.05 was used. All structure figures were generated using PyMOL (DeLano Scientific, San Carlos, California, USA). All structure have been deposited to Protein Data Bank.

## Additional Information

**How to cite this article**: Edlund, P. *et al*. The room temperature crystal structure of a bacterial phytochrome determined by serial femtosecond crystallography. *Sci. Rep.* **6**, 35279; doi: 10.1038/srep35279 (2016).

## Figures and Tables

**Table 1:** Data Collection and Refinement Statistics.

	LOWT PAS-GAF, ESRF	SFX PAS-GAF, LCLS	SACLA PAS-GAF, SACLA
PDB Code	5K5B	5L8M	5LBR
Data Collection			
Collection Temperature	100 K	293 K	293 K
Space group	C121	C121	C121
Cell dimensions			
a, b, c (Å)	93.76, 54.28, 70.15	94.10, 54.80, 69.90	96.22 55.49 71.63
α, β, γ (°)	90.00, 92.20, 90.00	90.00, 92.60, 90.00	90.00 92.84 90.00
Resolution (Å)	46.97–1.35 (1.38–1.35)†	38.20–2.10 (2.15–2.10)	71.55–2.20 (2.28–2.20)
R_merge_ (%)	3.60 (56.0)	—	—
R_split_ (%)	N/A	9.19 (85.43)	11.9 (67.7)
I/σI	18.37 (2.31)	7.77 (1.80)	5.62 (0.68)
CC(1/2)	99.9 (76.5)	98.0 (49)	97.6 (60.4)
Completeness (%)	98.07 (95.69)	99.97 (99.8)	100 (100)
Redundancy	4.36 (4.23)	819 (526)	81.2 (24.1)
Number of hits	N/A	114409	30146
Number of indexed hits	N/A	90428	20014
Refinement			
Resolution (Å)	39.37–1.35 (1.39–1.35)	38.20–2.10 (2.15–2.10)	71.55–2.20 (2.26–2.20)
Number of reflections	72086 (5211)	18732 (1202)	18389 (1326)
R_work_/R_free_	0.141/0.171 (0.260/0.288)	0.176/0.218 (0.299/0.297)	0.182/0.203 (0.496/0.483)
Number of atoms	2720	2505	2492
Average B factor (Å^2^)	25.47	42.92	50.26
R.m.s. deviations			
Bond lengths (Å)	0.010	0.007	0.008
Bond angles (°)	1.506	1.313	1.287

^†^Highest resolution shell is shown in parenthesis.
